# Autonomic and facial electromyographic responses to watching eyes

**DOI:** 10.1177/20416695231226059

**Published:** 2024-01-22

**Authors:** Tingji Chen, Terhi M. Helminen, Samuli Linnunsalo, Jari K. Hietanen

**Affiliations:** Department of Psychology, School of Education, 12582 Soochow University, Suzhou, Jiangsu, China; Human Information Processing Laboratory, Faculty of Social Sciences/Psychology, 7840 Tampere University, Tampere, Finland

**Keywords:** eye contact, eye tracking, facial electromyography, mutual gaze, skin conductance, heart rate

## Abstract

We measured participants’ psychophysiological responses and gaze behavior while viewing a stimulus person's direct and averted gaze in three different conditions manipulating the participants’ experience of being watched. The results showed that skin conductance responses and heart rate deceleration responses were greater to direct than averted gaze only in the condition in which the participants had the experience of being watched by the other individual. In contrast, gaze direction had no effects on these responses when the participants were manipulated to believe that the other individual could not watch them or when the stimulus person was presented in a pre-recorded video. Importantly, the eye tracking measures showed no differences in participants’ looking behavior between these stimulus presentation conditions. The results of facial electromyography responses suggested that direct gaze elicited greater zygomatic and periocular responses than averted gaze did, independent of the presentation condition. It was concluded that the affective arousal and attention-orienting indexing autonomic responses to eye contact are driven by the experience of being watched. In contrast, the facial responses seem to reflect automatized affiliative responses which can be elicited even in conditions in which seeing another's direct gaze does not signal that the self is being watched.

Seeing another person's direct gaze, i.e., gaze directed toward the self, can have a strong impact on an observer. Studies have shown the effects of other individuals’ direct gaze on affect and on many cognition-related processes, such as attention, memory, prosocial behavior, and self-awareness (for reviews, see Conty, George & Hietanen, 2016; Hamilton, 2016; Hietanen, 2018; Senju & Johnson, 2009). It has been postulated that some of these effects are due to the “watching eyes” effect (Conty et al., 2016; Pfattheicher & Keller, 2015); perception of another individual's direct gaze informs the observer that the self is attended to and watched by this individual.

Although many of the eye contact effects can be brought about by showing images of faces with a direct gaze, or even images showing just the eye region, there are studies which have shown that only a “live” person's gaze direction can evoke these effects. This seems to apply especially to a variety of psychophysiological responses. Several studies have shown that seeing a person's direct gaze elicits greater skin conductance responses (SCR; Helminen et al., 2011; Hietanen et al., 2008, 2020; Jarick & Bencic, 2019; Nichols & Champness, 1971; Pönkänen, Peltola, et al., 2011; Prinsen & Alaerts, 2019), greater evoked visual brain responses (Pönkänen, Alhoniemi, et al., 2011), and increased electroencephalographic, relative left-sided frontal alpha activity associated with positive affect and motivational approach tendency (Hietanen et al., 2008; Pönkänen, Peltola, et al., 2011; Uusberg et al., 2015) compared to seeing a person's averted gaze when the person is presented live, whereas no effect of gaze direction is observed when the same faces are shown as still images (Hietanen et al., 2008; Pönkänen, Alhoniemi, et al., 2011; Pönkänen, Peltola, et al., 2011) or pre-recorded videos (Hietanen et al., 2020; Lyyra et al., 2018; Prinsen & Alaerts, 2019) in otherwise comparable experimental conditions. The results of these studies have suggested that another individual's gaze direction has an effect on these responses only when the observer knows that they are being looked at by another mind (e.g., Hietanen et al., 2008). This sort of mentalizing does not occur, at least not to the same degree, when an observer is looking at an image.

More direct evidence for the role of “being watched” explaining the observed differences in physiological reactions in response to gaze stimuli presented in live versus pictorial format was gained from a study in which participants’ belief of whether the live (stimulus) person was able to see them or not was directly manipulated (Myllyneva & Hietanen, 2015). In one condition, the participant and the stimulus person were able to see each other normally, whereas in the other condition, the participant was led to believe that the vision from the other person's side was blocked with a one-way window. The results showed that the other person's direct gaze elicited enhanced autonomic (skin conductance and heart rate deceleration) and brain (frontal P3 event-related potential) responses only when the participants knew that the other person could see them, but not when they believed that the other person could not see them. Additional evidence for the “being watched” explanation was gained from a study in which autonomic arousal responses were measured in live interaction, in a bidirectional video call, and during watching a mere video (Hietanen et al., 2020). Autonomic arousal responses were greater to direct than averted gaze in live interaction and bidirectional video call, but not when the participants were watching a mere video of the other person.

Interestingly, in the study mentioned just above (Hietanen et al., 2020), the authors also measured facial electromyography (EMG) responses. The results showed greater zygomatic (cheek region) responses to direct versus averted gaze indicative of positive affect, replicating previous findings (Hietanen et al., 2018), but, unlike the other psychophysiological responses described above, the zygomatic responses discriminated between the direct and averted gaze in all viewing conditions. Similar results were found in another recent study in which the same “belief of being watched” (BW) manipulation as described above was used (Kiilavuori et al., 2022). The zygomatic responses discriminated between the direct and averted gaze independent of whether the participants believed to be watched or not.

The present study had two main aims. First, we wanted to investigate and compare, and hopefully replicate, the effects of gaze direction on three different psychophysiological measures (SCR, heart rate deceleration responses, and facial EMG responses) in three different viewing conditions manipulating the observers’ experience of being watched. By investigating all these effects in a single experiment, we hoped to gain a more robust understanding of how different types of being watched manipulations influence autonomic and facial responses.

The second aim of this study was to investigate participants’ looking behavior in different stimulus presentation conditions and whether the abovementioned eye contact effects on the psychophysiological responses in different presentation conditions could be explained by differences in overt visual attention deployment. Even though the participants were instructed to look toward the stimuli in all aforementioned studies, participants’ eye movements were recorded only in the study by Prinsen and Alaerts (2019). The results showed that the stimulus person's gaze direction did not have an effect on the average fixation duration, but participants made more fixations toward the eye region when the gaze was direct in comparison to when gaze was averted. Importantly, the average duration of fixations toward the eye region did not differ between the live and video presentation conditions. Thus, the study by Prinsen and Alaerts (2019) provided evidence that the differential gaze direction effects on autonomic arousal responses in live versus video conditions were not due to differences in the participants’ looking behavior.

However, there are other findings which suggest that the presentation mode of social stimuli may have an effect on attention. For example, an eye-tracking study showed that although participants frequently looked toward a person appearing on a television screen in a waiting room, they looked away and thus avoided potential eye contact when facing the same person live in the waiting room (Laidlaw et al., 2011). Another study using eye tracking with a simultaneous video recording of the scene during a walk revealed that pedestrians close to the walker were fixated less in the real world than when seen on the videos viewed later in the laboratory (Foulsham et al., 2011). Similar results have been reported in studies in which the participants viewed pre-recorded videos but some of the participants were told that they would be presented a “live” video-feed while others were told that they would be viewing pre-recorded videos (Gregory & Antolin, 2019; Gregory et al., 2015; Holleman et al., 2020). These kinds of results have been explained by the influence of social norms of avoiding eye contact with strangers and the associated social risks if these norms are violated (Gregory & Antolin, 2019; Laidlaw et al., 2011). These eye-tracking studies suggest that attention is allocated very differently on people in real life versus pictures and that emotional and social factors may exert a strong influence on attentional processes.

There is also evidence that fixation to the eye region can have an influence on the activation of the social brain network during face perception. In an fMRI study, participants were presented with emotional face stimuli in two conditions: in a free-viewing mode and when a fixation cross was added in the eye region (Hadjikhani et al., 2017). The results showed that the brain activation, for example, in fusiform gyrus, posterior superior temporal sulcus, and dorsomedial prefrontal cortex, and the amygdala connectivity with other areas of the social brain were greater when the participants’ attention was constrained to the eye region. Taken together, the existing evidence suggests that the presentation mode might have an impact on the allocation of attention to faces, and this might affect the activation of the social brain and, further, modulate the psychophysiological responses. Thus, we considered it important to investigate further whether the observed differences in psychophysiological responses to other individuals’ direct and averted gaze between live and image presentation conditions could be related to differences in allocation of participants’ overt attention.

For these two aims, in the present study, we measured participants’ psychophysiological responses and gaze behavior, during viewing a stimulus person's direct and averted gaze in three different conditions. The same stimulus person appeared in all conditions. In the first condition, a live stimulus person was presented through a smart window (liquid crystal [LC] window). In this condition, it was possible to make a genuine eye contact: when the participant was looking at the stimulus person and the stimulus person had a direct gaze (i.e., looking at the participant), the participant was aware that they both were watching each other. In the second condition, the stimulus person was also presented through a smart window, but the participant was led to believe that a one-way window was placed onto the smart window in such a way that the stimulus person could not see through. Thus, for the participant, the stimulus person appeared visually identical in this and the first condition, but the participant believed that the stimulus person could not see them. A similar procedure has been used in previous studies (Hietanen et al., 2019; Myllyneva & Hietanen, 2015). In the third condition, a video of the stimulus person was presented. Obviously, in this condition too, the participants were aware that a genuine eye contact was not possible.

Three types of physiological responses were measured: SCR, heart rate deceleration responses, and facial EMG responses from zygomaticus major (cheek region) and orbicularis oculi (periocular region) muscles. Sympathetic SCR index physiological arousal (Critchley, 2002), a central component of emotional reactions (Plutchik, 1980). Orienting of attention to external stimuli is accompanied by a deceleration of heart rate and the deceleration is amplified by affectively and motivationally salient stimuli (Bradley, 2009; Graham & Clifton, 1966; Lang & Bradley, 2010). We expected that the findings would replicate previous results (Hietanen et al., 2008; Myllyneva & Hietanen, 2015; Pönkänen, Peltola, et al., 2011) and show that the eye contact effect, that is, greater responses to direct than averted gaze, is observed only in the condition enabling a genuine eye contact. In addition to these autonomic nervous system responses, we also measured rapid facial EMG responses from the zygomatic and periocular regions. Increased zygomatic and periocular region muscle activity has been suggested to reflect an automatic positive affective reaction (Cacioppo et al., 1986; Dimberg, 1990; Larsen et al., 2003) although these responses, and especially the zygomatic response, can also reflect highly automatized affiliative reactions (Hess & Fischer, 2013; Knutson, 1996; Niedenthal et al., 2012; Parkinson, 2005). Based on the previous findings (Hietanen et al., 2018, 2020; Hietanen & Peltola, 2021; Kiilavuori et al., 2022), we expected that the zygomatic and periocular EMG responses would be greater to seeing another's direct than averted gaze irrespective of the stimulus presentation condition.

## Methods

### Participants

Twenty-eight participants (15 females; age range, 20–34 years; mean age 23.3 years) with normal or corrected-to-normal vision were recruited via mailing lists of Tampere University. According to a power analysis performed with MorePower 6.0 software (1–β = .80, α = .05; Campbell & Thompson, 2012), this sample size was adequately powered to detect a medium to large effect size (η*_p_*^2^ = .16) in a 2 × 3 repeated-measures analysis of variance (ANOVA). Similar sample sizes were reported in the previously related studies (e.g., Myllyneva & Hietanen, 2015; Prinsen & Alaerts, 2019). All participants provided a written, informed consent, and received course credits or a movie ticket for their participation. After data collection, two participants had to be excluded because of unsuccessful mirror deceit (see below) and one participant was excluded due to technical errors in the measurements. Additionally, seven participants were excluded from the periocular region EMG analyses due to a high number of signal artifacts. Finally, for the eye tracking data analyses, one participant's data were missing and three participants were excluded due to missing data in all trials for some of the conditions. Hence, the final data sample consisted of 25 participants for the SCR, zygomatic EMG, and heart rate (HR) data analyses, 18 participants for the periocular EMG analyses, and 21 participants for the eye tracking analyses. Ethical statement for the study was obtained from the Ethics Committee of the Tampere region and the study conformed to the Declaration of Helsinki.

### Stimuli

**Figure 1.**
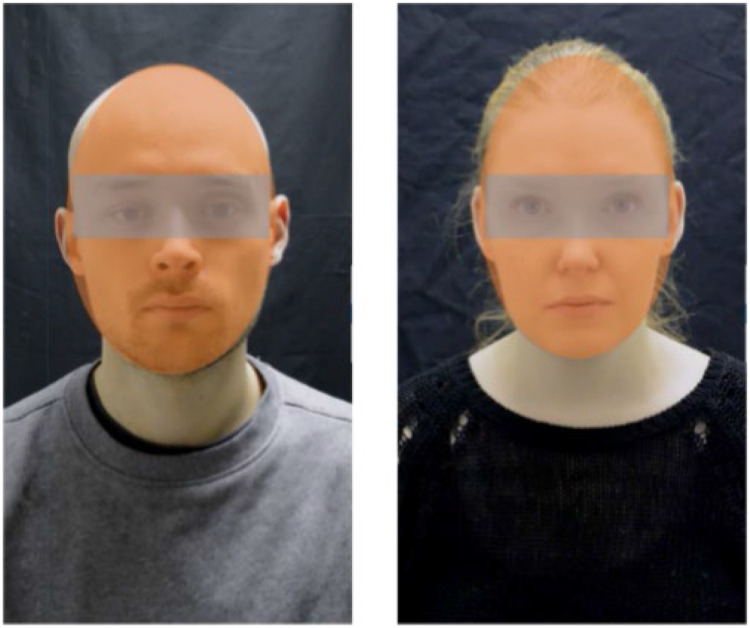
Examples of the stimuli. The photographs also illustrate the AOI for the eye-tracking analyses. The gray area represents the AOI for the eye region and the orange area represents the AOI for the face region analyses.

*Note.* AOI = areas of interest.

One female and one male served as stimulus persons (models) seen against a black background (Figure 1). Each participant was presented with one model of the same gender. The participants’ and models’ gender was matched in order to avoid any impact by the matching versus non-matching gender. During the trials, the model bore a neutral expression and was instructed to blink naturally when necessary but to keep the face otherwise as motionless as possible. In half of the trials, their gaze direction was direct and in the other half of the trials, the gaze direction was averted (equal number of trials with gaze averted 50° to the left and right). When preparing the video stimuli, the instructions and settings (background, clothes, hair style, etc.) were the same as in the live conditions. Both in the live conditions and in the video condition, the stimuli (models’ faces) were presented through a 30 × 40 cm voltage-sensitive LC shutter (NSG UMU Products Co., Ltd.). The shutter was fixed to a large black panel and positioned on the table between the model and the participant. The LC shutter switched between opaque and transparent states within an overall speed of 3 ms and the state of the shutter (transparent or opaque) was operated by E-Prime 2.0 software (Psychology Software Tools, Pittsburgh, PA) running on a desktop computer. In the live conditions, the models were seated on the other side of the table at a distance of 40 cm from the LC shutter. On the other side of the LC shutter, the participants placed their heads on a chin rest at a distance of 40 cm from the shutter. In the video condition, a computer screen was placed behind the LC shutter in a portrait orientation in such a way that the participants would not see the frame of the screen. The model's face on the screen was life-size. For each participant, their seat was adjusted in such a way that the model's eyes (in the live and in the video conditions) were vertically at the same level with the participant's eyes.

### Procedure

Upon a participant's arrival, the experimenter explained to the participant that the purpose of the experiment was to measure their physiological and behavioral responses in a simple interaction situation. In order to avoid increased self-awareness of one's own looking behavior or facial expressions, the participants were deceived to believe that the purpose of the eye tracking glasses was to measure changes in the pupil size, and the purpose of the facial electrodes was to measure skin temperature. They were told that there would be three separate blocks in the experiment. After this, the electrodes were attached. Separate instructions were given prior to each condition. In the condition of BW, the participants were introduced the panel with the LC shutter and informed that when the shutter is open, they and the model person can see each other. In the condition of belief of not being watched (BnW), a deception procedure was applied (for a detailed description of this deception, see Myllyneva & Hietanen, 2015). After introducing the LC shutter, the experimenter told participants that, in this condition, a one-way window would be slid on the LC shutter so that they could still see the model person, but the model person could not see them. In reality, the model slid a transparent plexi sheet to cover the LC shutter on their side. In the video condition, the participants were told that they would be seeing a video of the same model person as in the other conditions on the screen that was placed on the other side of the LC shutter. In all conditions, the participants were instructed that their task was simply to look at the model person, while the LC shutter was transparent.

After giving the instructions, the participant was asked to put on the eye tracking glasses (SensoMotoric Instruments) and place their head on a chin rest. Before starting the experiment, the experimenter calibrated the eye-tracking glasses with a one-point calibration. Calibration was done by asking the participant to focus on a calibration target (a black asterisk) drawn on a big paper sheet that was placed in the middle of the LC shutter.

The experiment had a 3 (presentation condition: BW, BnW, video) × 2 (gaze: direct, averted) design. The three presentation conditions were separated into three blocks and the sequence of the three conditions was balanced across the participants. Each block included six trials of direct gaze, three trials of left-averted gaze, three trials of right-averted gaze, and two trials for calibration. The order of the gaze direction trials was randomized and after each four trials, there was one trial for calibration. Each trial lasted for 5,000 ms during which the shutter was open. The inter-trial interval (from offset to onset) was manually controlled by the experimenter, and it varied between 20 s and 45 s. The experimenter started the next trial when the participant's skin conductance returned to baseline level.

### Measures and Data Reduction

#### Psychophysiological Measures and Data Reduction

The SCR was measured using two silver–silver chloride electrodes attached to the palmar surface of the medial phalanxes of the index and middle fingers of the participant's left hand. Skin conductance was recorded with the BrainVision Recorder software (Brain Products GmbH, Munich, Germany). Offline, the data were re-sampled to 100 Hz and filtered with a 10 Hz low-pass by the BrainVision Analyzer 2.1 software. The SCR response was defined as a maximum amplitude change from the baseline level (at the stimulus onset) during a time window of 5 s, which started 1 s after the stimulus onset. The trials with slight amplitude changes (less than 0.01 µS) were coded as zero response trials. To take both the response strength and frequency into account, trials with zero responses were also included to compute the magnitude of SCR for each condition (Christopoulos et al., 2019; Dawson et al., 2000). Trials with amplitude rise of over 0.01 µS during the first second after the stimulus onset were rejected from the analysis. Based on this criterion and technical errors, 10.34% of the trials were excluded. A logarithmic transformation [log (SCR + 1)] was applied to correct for non-normal distribution.

Electrocardiogram (ECG) was recorded with bipolar electrodes attached to the left and right forearm and one extra electrode on the forehead as a ground. Offline, R-peaks were identified by an in-house, Matlab-based algorithm and manually checked and corrected when R-peaks were falsely detected and missed. Then the signal was segmented into 5,500-ms epochs with 500 ms prior to the stimulus onset as baseline. Segments with excessive distortion in the ECG signal (2%) were excluded. For the accepted segments, inter-beat intervals (IBIs; i.e., the time intervals between two successive R-peaks) were converted to beats per minute (BPM) in 500-ms intervals. The analyses were performed using HR change scores, which were calculated by subtracting the baseline BPM during the 500 ms preceding the stimulus onset from each of the BPMs during the subsequent 500-ms intervals after the stimulus onset. Thus, positive HR change scores indicated HR acceleration and negative scores indicated HR deceleration.

EMG was recorded with bipolar electrodes, which were attached on the participant's left cheek (zygomaticus major) and outer corner of the left eye (orbicularis oculi) according to the guidelines by Fridlund and Cacioppo (1986). The raw signal was filtered with a 28–249 Hz band-pass filter and rectified offline by using the BrainVision Analyzer 2.1 software. The signal was visually inspected and trials with excessive baseline muscle activity or eye blinks were discarded as artifacts (1.78% of the trials for zygomaticus data, 18.52% of the trials for periocular data). Participants with less than three included trials in any condition were excluded from the analyses of the respective muscle area (7 participants for periocular muscle area). The data from included trials were averaged within each participant, stimulus condition, and gaze condition and segmented into 3,500-ms epochs with 500 ms prior to the stimulus onset as baseline. The segments were then converted to standardized Z scores to reduce the influence of extreme values. For statistical analyses, the muscle activity values were calculated for each participant, muscle region, stimulus condition, and gaze condition by subtracting the baseline value from their respective post-stimulus epoch.

#### Eye Tracking Data

Binocular eye position was monitored and recorded using eye tracking glasses (SMI ETG) with a sampling rate of 30 Hz. The glasses had a built-in scene camera which records the participant's field of view about 50° (horizontal) × 40° (vertical) with the point of regard superimposed over the image as a circular cursor in the recorded video. The recorded video allowed frame-by-frame analysis of the gaze direction. Using the SMI BeGaze software, the areas of interest (AOIs) were drawn frame by frame to measure the total fixation dwell times to the face and eye region of the models in each condition. The face region was defined as the entire face of the model (see Figure 1). The eye region covered horizontally an area between the left and right outline of the face and vertically an area between the inner (upper) edges of the eyebrows and the halfway of the nasal bridge. The dependent variables of interest were the total dwell time of the face and eye region AOI for the three presentation conditions and the two gaze direction conditions. We also calculated the proportional eye region dwell time, that is, the total dwell time to the eye region as a proportion of the total dwell time to the face region.

### Data Analysis

The eye tracking, SCR, and EMG analyses were conducted using a 3 (presentation condition: BW vs. BnW vs. video) × 2 (gaze: direct vs. averted) repeated-measures ANOVA. The HR data were analyzed with a 3 (presentation condition) × 2 (gaze) × 10 (time) repeated-measures ANOVA. Planned comparisons were performed for the analyses of simple main effects when interactions were observed. A Greenhouse–Geisser correction was applied when appropriate. All statistical analyses were performed using the SPSS package.

## Results

### Skin Conductance

**Figure 2.**
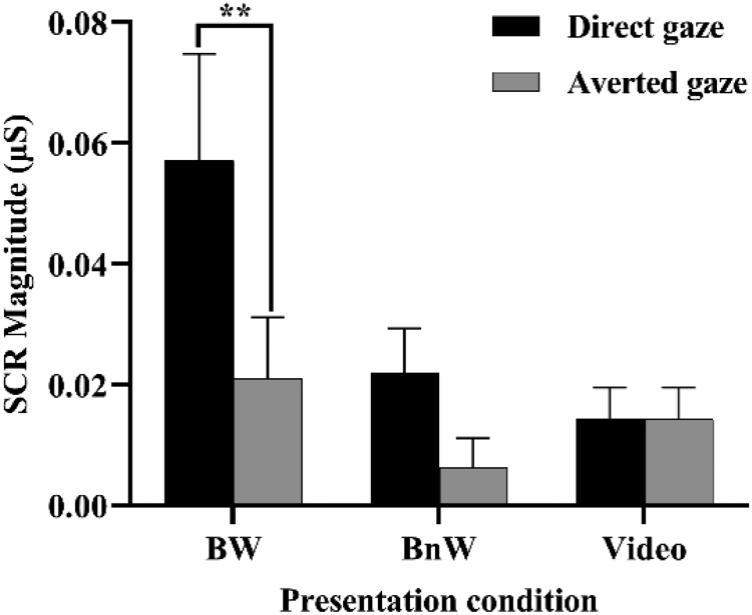
Skin conductance responses (mean + *SEM*) to direct and averted gaze in different presentation conditions.

*Note. SEM* = standard error of the mean. ** *p* < .01.

The analysis of the SCR data (Figure 2) showed a significant main effect of gaze, *F*(1, 24) = 7.665, *p* = .011, η*_p_*^2 ^= .242. Overall, the SCR responses were greater in response to direct gaze (0.030 µS ± 0.007) than to averted gaze (0.014 µS ± 0.005). There was also a significant main effect of presentation condition, *F*(2, 48) = 4.022, *p* = .024, η*_p_*^2^*^ ^* = .144. Post hoc comparisons (LSD) showed that the SCRs in the BW condition (0.039 µS ± 0.013) were significantly greater than those in the video condition (0.012 µS ± 0.005; *p* = .032, *d* = 0.507). Also, there was a trend for greater SCRs in the BW condition than in the BnW condition (0.014 µS ± 0.004, *p* = .066, *d* = 0.473). Importantly, there was a statistically significant interaction between presentation condition and gaze, *F*(2, 48) = 4.233, *p* = .02, η*_p_*^2^*^ ^* = .15. Paired *t*-tests indicated significantly greater SCRs to direct (0.057 µS ± 0.018) versus averted (0.021 µS ± 0.010) gaze in the BW condition, *t*(24) = 2.822, *p* = .009, *d* = 0.564, but, in the BnW, *t*(24) = 1.742, *p* = .094, *d* = 0.348, and video condition, *t*(24) = −0.683, *p* = .501, *d* = −0.137, there were no statistically significant differences between the SCRs in response to direct versus averted gaze.

### Heart Rate

**Figure 3.**
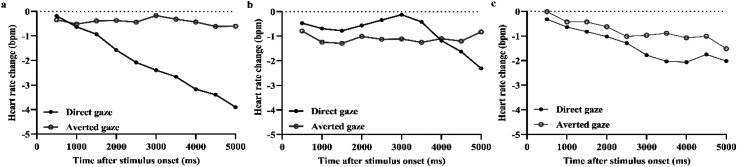
Heart rate changes in response to direct and averted gaze in the (a) BW condition, (b) BnW condition, and (c) video condition.

*Note.* BW = belief of being watched; BnW = belief of not being watched.

A 3 × 2 × 10 within-subjects ANOVA showed a main effect of time, reflecting a HR deceleration, *F*(9, 216) = 6.618, *p* = .001, η*_p_*^2^*^ ^* = .216. The main effects of presentation condition, *F*(2, 48) = 0.187, *p* = .830, η*_p_*^2^*^ ^* = .008, and gaze, *F*(1, 24) = 3.005, *p* = .096, η*_p_*^2^*^ ^* = .111, were not significant. However, there were significant two-way interactions between gaze and time, *F*(9, 216) = 4.004, *p* = .013, η*_p_*^2^*^ ^* = .143, and between presentation condition and time, *F*(18, 432) = 1.678, *p* = .040, η*_p_*^2 ^= .065, and a three-way interaction between presentation condition, gaze, and time, *F*(18, 432) = 1.649, *p* = .046, η*_p_*^2 ^= .064. Because of the interactions, we first conducted a two-way ANOVA for each presentation condition. There was a significant interaction between gaze and time in the BW condition, *F*(9, 216) = 4.572, *p* *<* .001, η*_p_*^2^*^ ^* = .16. The interactions were not significant in the BnW and video conditions, *F*(9, 216) = 1.736, *p* = .082, η*_p_*^2 ^= .067; *F*(9, 216) = 0.475, *p* = .890, η*_p_*^2 ^= .019. Furthermore, we analyzed the effect of gaze separately in the three presentation conditions. In the BW condition (see, Figure 3), there was a trend for greater HR deceleration for direct than averted gaze, *t*(24) = −1.982, *p* = .059, *d* = −0.396. There were no statistically significant differences between direct and averted gaze in the BnW condition, *t*(24) = 0.352, *p* = .728, *d* = 0.070, nor in the video condition, *t*(24) = −1.102, *p* = .281, *d* = −0.221.

### EMG Responses

**Figure 4.**
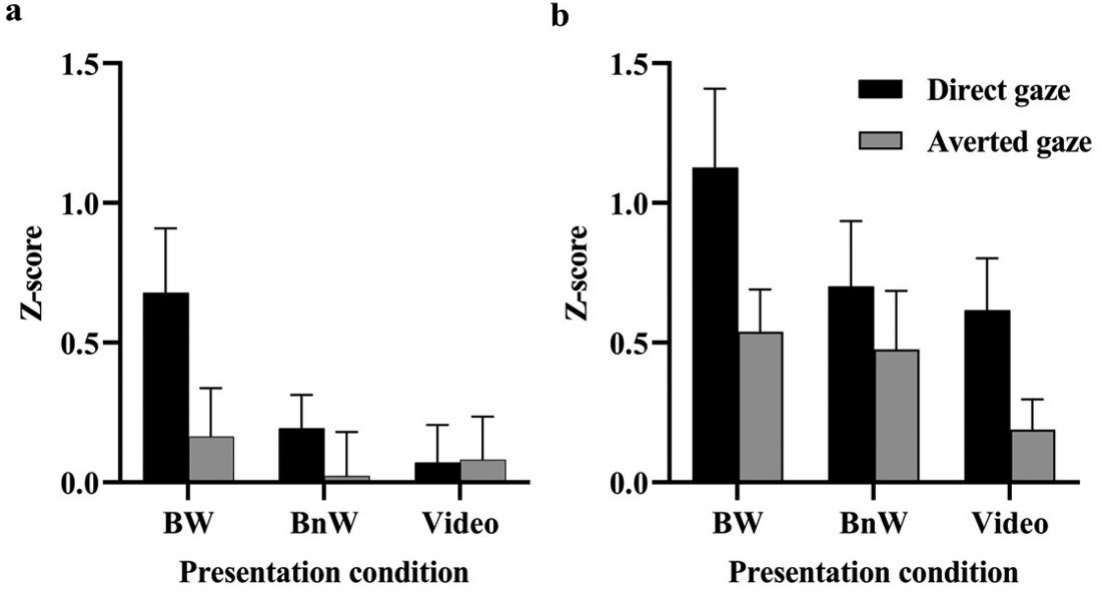
The (a) zygomatic and (b) periocular EMG responses (mean + *SEM*) to direct and averted gaze in different presentation conditions.

*Note.* EMG = electromyography; *SEM* = standard error of the mean.

For the zygomaticus region responses (Figure 4), a 3 × 2 ANOVA showed a main effect of gaze direction, *F*(1, 24) = 6.335, *p* = .019, η*_p_*^2^*^ ^* = .209, suggesting overall greater responses to direct gaze (0.315 ± 0.099) than to averted gaze (0.089 ± 0.087). The main effect of presentation condition, *F*(2, 48) = 1.800, *p* = .176, η*_p_*^2^*^ ^* = .070, and the Presentation Condition ×Gaze interaction, *F*(2, 48) = 2.285, *p* = .113, η*_p_*^2^*^ ^* = .087, were not statistically significant. For the periocular region, there was a main effect of gaze direction, *F*(1, 17) = 16.827, *p* = .001, η*_p_*^2^*^ ^* = .497; the responses were overall greater to direct gaze (0.815 ± 0.147) than to averted gaze (0.401 ± 0.114). Again, the main effect of presentation condition, *F*(2, 34) = 2.376, *p* = .108, η*_p_*^2^*^ ^* = .123, and the Presentation Condition × Gaze interaction, *F*(2, 34) = 0.501, *p* = .610, η*_p_*^2 ^= .029, were not statistically significant.

### Eye Tracking

**Figure 5.**
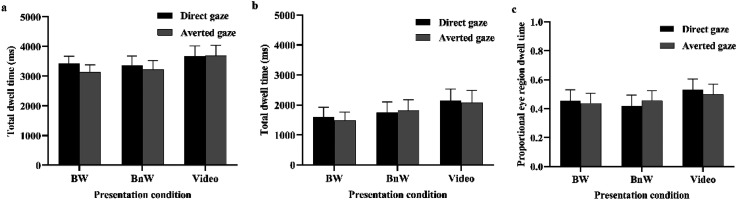
The total dwell times (mean + *SEM*) to (a) face region, (b) eye region, and (c) proportional eye region dwell times as a function of presentation condition and gaze direction.

*Note. SEM* = standard error of the mean.

The analysis of the total dwell times on face region (Figure 5a) showed that there was a trend, *F*(1, 20) = 3.927, *p* = .061, η*_p_*^2^*^ ^* = .164, for longer total dwell times for direct gaze (3,491 ms ± 216, *M* ± *SE*) than for averted gaze (3,351 ms ± 193). The main effect of presentation condition, *F*(2, 40) = 0.779, *p* = .466, η*_p_*^2^*^ ^* = .037, or the interaction between presentation condition and gaze, *F*(2, 40) = 0.984, *p* = .383, η*_p_*^2^*^ ^* = .047, were not significant. The analysis of the dwell times on the eye region did not show any statistically significant main or interaction effects (presentation condition, *p* = .448; gaze, *p* = .686; Presentation Condition × Gaze, *p* = .680; Figure 5b). The analysis of the proportional eye region dwell times did not show any statistically significant main or interaction effects either (presentation condition: *p* = .680; gaze: *p* = .867; Presentation Condition × Gaze: *p* = .360; Figure 5c).

## Discussion

In the present study, we investigated participants’ autonomic and facial responses to another person's direct (eye contact) and averted gaze in three different conditions: both seeing each other normally, participants seeing the other person normally but believing that this person cannot see them (because of an alleged one-way window), and participants seeing a video of the person. We measured, in all conditions, participants’ gaze behavior with head-mounted eye-tracking glasses as well as SCR (autonomic arousal), heart rate deceleration responses (attention orienting), and electromyographic responses from the zygomatic and periocular muscle regions (affective/affiliative reactions). We were interested in replicating previous findings indicating that another person's direct gaze (in comparison to averted gaze) elicits greater autonomic (skin conductance and heart rate) responses than averted only in the conditions when the participants have an experience of being seen by the other person. In other words, we wanted to show again that the psychophysiological responses are not responses to other individual's eyes and gaze direction as such, but responses to watching eyes. Instead, for the facial EMG responses, previous studies had indicated greater responses to direct versus averted gaze irrespective of the participants’ experience of being watched, and we wanted to see whether this result is also replicated. By including both autonomic and facial responses as well as the three viewing conditions into the same experiment, we aimed to a robust experiment to investigate how different types of being watched manipulations influence autonomic and facial responses. Importantly, we also aimed to investigate whether the possible differences in the effects of another's gaze direction between the different viewing conditions could be due to differences in participants’ looking behavior during stimulus presentation.

Regarding the effect of watching eyes, the results showed that the SCRs were statistically significantly greater to direct than averted gaze only in the condition in which the participants had the experience of being watched by the other individual. Compatibly, the heart rate deceleration response was numerically greater to direct than averted gaze only in this condition, although the difference was only trending and did not reach statistical significance (*p* = .059). For both the SCR and heart rate deceleration responses, the gaze direction had no statistically significant effects in the condition in which the participants were manipulated to believe that the other individual could not see them (BnW condition) nor in the condition in which it was physically impossible for the social stimulus to watch the participant (pre-recorded video condition). The SCR results replicate previous findings showing the eye contact effect in bidirectional viewing conditions with a live person or in a video call, but not when the person appears in an image or in a video (Hietanen et al., 2008, 2020; Pönkänen, Peltola et al., 2011; Prinsen & Alaerts, 2019) or when the participants are manipulated to believe that a live person cannot see the participant (Myllyneva & Hietanen, 2015). The results regarding the effect of the stimulus presentation condition on the heart rate deceleration responses also replicate previous findings (i.e., BW vs. BnW condition; Myllyneva & Hietanen, 2015). In all, these results showed that the effect of the eye contact on the autonomic responses was not due to the visual appearance of another individual's eyes and gaze direction as such and that the effect was insensitive to the physical presence of the other individual, but that the critical ingredient was the experience of being watched.

As expected, the EMG results differed from those of the autonomic responses. Replicating previous results (e.g., Hietanen et al., 2018, 2020; Hietanen & Peltola, 2021; Kiilavuori et al., 2021, 2022) both the zygomatic and periocular EMG responses were greater to direct than averted gaze. However, this was seen irrespective of presentation condition: the results showed no statistically significant interaction between gaze direction and presentation condition. These findings are also compatible with previous results showing that the zygomatic responses discriminated between the direct and averted gaze independent of whether the other person was seen live through the LC window (like in the present study), or on the computer screen during a bidirectional video call, or online on the computer screen when the other person's webcam was removed and the observers knew that the other individual could not see them (Hietanen et al., 2020). In another previous study (Kiilavuori et al., 2022), the authors used the same BW and BnW stimulus presentation conditions as in the present study, and the results of that study also showed zygomatic discrimination between direct and averted gaze in both conditions. These results provide accumulating evidence that the facial reactions are not be sensitive to the top-down influence of being watched, at least not to the same degree, as compared to the autonomic SCRs and heart rate deceleration responses.

In general, the zygomatic and periocular responses to eye contact may reflect either a positive affective (emotional) reaction or communication of affiliation (Hess, 2021; Hess et al., 2000; Martin et al., 2017; Niedenthal et al., 2012). An emotional response is considered to include both the facial muscle activity and enhanced autonomic activation preparing the body for action (cf. Plutchik, 1980). The present results showed that while, in the BW condition, seeing another person's direct gaze enhanced zygomatic and periocular activity as well as autonomic arousal, in the BnW and in the video condition, these responses seemed dissociated; zygomatic and periocular activity was observed but without enhanced autonomic arousal. Therefore, this finding suggests that the facial reactions in the present study did not reflect an affective reaction, but rather an affiliative reaction. In facial mimicry research (i.e., facial responses to other people's facial expressions), it has been reported that especially the smiling responses are resistant to contextual effects because they do not involve personal costs and are socially acceptable due to their affiliative nature (Bourgeois & Hess, 2008; Künecke et al., 2017). It is possible that, because of this, the present results showed that affiliative smiling responses were triggered relatively automatically in response to another's direct gaze regardless of whether they could serve a communicative function or not.

The eye tracking results revealed no differences in participants’ looking behavior between the stimulus presentation conditions. The participants allocated attention on the eye region and on the face region similarly in each condition. Thus, the present study shows that the differential eye contact effects on the psychophysiological responses between the stimulus conditions are not due to differences in participants’ looking behavior. This result replicates findings from a previous study showing differential gaze direction effects on autonomic arousal responses in live and video presentation conditions, but no differences in the gazing behavior (Prinsen & Alaerts, 2019). The total dwell times on the face region showed a trend for an effect of gaze direction; the participants allocated their attention longer for faces with direct gaze than for faces with averted gaze. This finding is compatible with those from previous studies (e.g., Palanica & Itier, 2012; Prinsen & Alaerts, 2019). In the present study, the dwell times on the eye region did not differ between the stimulus conditions. This indicates that a person's direct gaze increased observers’ attention allocation toward the person's whole face area and not just the eye region.

In contrast to the present results, previous studies have shown that the presentation conditions can have an effect on attention allocation toward faces. Previous studies have shown that less overt attention is allocated toward other individuals when they are (or believed to be) capable of seeing the observer as compared to when this is not the case, for example, when seeing others in a video recording (Foulsham et al., 2011; Holleman et al., 2020; Laidlaw et al., 2011). A very likely explanation for this difference is that observers allocate their attention on other persons differently when they are presented shortly and repeatedly as a “stimulus” (as in the present study, only 5 s at a time), as compared to when they are seen in more natural conditions, allowing the participants to view them considerably longer (e.g., 30 s in the study by Holleman et al., 2020).

Although we aimed to a similar sample size which has been reported in the previous studies, for example, 26 participants in Myllyneva and Hietanen (2015) and 23 participants in Prinsen and Alaerts (2019), a number of participants had to be removed from the final data analyses due to different reasons, and therefore the sample size fell short of the intended. This is a limitation of the present study. This concerns especially the reliability of the EMG results. Although the statistical analysis did not reveal a significant interaction between gaze direction and stimulus presentation condition for the zygomatic responses, inspection of the mean results (see Figure 5a) shows that, in the video condition, the mean zygomatic response was not greater to direct versus averted gaze. Thus, this would suggest that this response could also be sensitive to the experience of being watched. With a greater sample size, we could have tested more reliably whether the zygomatic response is sensitive to the watching eyes effect or not. Having said this, we want to remind that there are previous results, as cited above, which also point to the direction that zygomatic responses are not modulated by the experience of being watched, and, moreover, the periocular responses of the present study also showed insensitivity for this modulation. Therefore, despite this limitation, the current evidence seems to point to the direction that the facial reactions are differently sensitive to the top-down influences by the experience of being watched as compared to responses of the autonomic nervous system.

In conclusion, by including three different stimulus presentation conditions in one experiment, the present study provided further evidence that the effects of eye contact on autonomic responses indexing affective arousal (SCR) and attention orienting (heart rate deceleration response) are conditional to the experience of being watched. The visual perception of another individual's direct gaze is not enough to trigger these physiological responses but requires the experience of being watched. Importantly, the present study showed that the presence of the eye contact effect is not conditional to observers’ looking behavior as the eye tracking results indicated that the other individual was gazed at similarly independent of whether the viewing condition allowed bidirectional visibility—and the experience of being watched—or not. Interestingly, the results also provided accumulating evidence that an affiliative smiling response to seeing another's direct gaze may be such an automatic response that is released even in conditions in which the eyes are not really watching, that is, in conditions in which reciprocal sending and receiving direct gaze, a genuine eye contact, is not possible.
